# Benralizumab in Patients With Severe Eosinophilic Asthma With and Without Chronic Rhinosinusitis With Nasal Polyps: An ANANKE Study *post-hoc* Analysis

**DOI:** 10.3389/falgy.2022.881218

**Published:** 2022-05-18

**Authors:** Maria D'Amato, Francesco Menzella, Elena Altieri, Elena Bargagli, Pietro Bracciale, Luisa Brussino, Maria Filomena Caiaffa, Giorgio Walter Canonica, Cristiano Caruso, Stefano Centanni, Fausto De Michele, Fabiano Di Marco, Elide Anna Pastorello, Girolamo Pelaia, Paola Rogliani, Micaela Romagnoli, Pietro Schino, Gianenrico Senna, Alessandra Vultaggio, Alessandra Ori, Lucia Simoni, Silvia Boarino, Gianfranco Vitiello, Maria Aliani, Stefano Del Giacco

**Affiliations:** ^1^UOSD Malattie Respiratorie “Federico II”, Ospedale Monaldi, AO Dei Colli, Napoli, Italy; ^2^UOC Pneumologia, Ospedale “S. Valentino”, Montebelluna (TV) - AULSS 2 Marca Trevigiana, Montebelluna, Italy; ^3^Reparto di Pneumologia, P.O. Garbagnate Milanese, Garbagnate Milanese, Italy; ^4^Respiratory Diseases and Lung Transplantation, Department of Medical and Surgical Sciences & Neurosciences, Siena University Hospital, Siena, Italy; ^5^Reparto di Pneumologia, Ospedale Ostuni, Ostuni, Italy; ^6^Dipartimento di Scienze Mediche, SSDDU Allergologia e Immunologia Clinica, Università degli Studi di Torino, AO Ordine Mauriziano Umberto i - Torino, Torino, Italy; ^7^Cattedra e Scuola di Allergologia e Immunologia Clinica, Dipartimento di Scienze Mediche, Università di Foggia, Foggia, Italy; ^8^Department of Biomedical Sciences, Humanitas University, Pieve Emanuele, Italy; ^9^Asthma & Allergy Unit-IRCCS Humanitas Research Hospital, Rozzano, Italy; ^10^Dipartimento di Scienze Mediche e Chirurgiche, Fondazione Policlinico A. Gemelli, IRCCS, Università Cattolica del Sacro Cuore, Rome, Italy; ^11^Respiratory Unit, ASST Santi Paolo e Carlo, Department of Health Sciences, Università degli Studi di Milano, Milano, Italy; ^12^UOC Pneumologia e Fisiopatologia Respiratoria, AORN A. Cardarelli, Napoli, Italy; ^13^Department of Health Sciences, University of Milan, Pneumology, ASST Papa Giovanni XXIII, Bergamo, Italy; ^14^Allergy & Immunology, Niguarda Hospital, Milano, Italy; ^15^Dipartimento di Scienze della Salute, Università Magna Graecia, Catanzaro, Italy; ^16^Division of Respiratory Medicine, University Hospital “Tor Vergata”, Rome, Italy; ^17^Unit of Respiratory Medicine, Department of Experimental Medicine, University of Rome “Tor Vergata”, Rome, Italy; ^18^UOC Pneumologia, ULSS 2 Marca Trevigiana, Treviso, Italy; ^19^Fisiopatologia Respiratoria, Ospedale Generale Regionale, Ente Ecclesiastico “F. Miulli”, Acquaviva delle Fonti, Italy; ^20^Department of Medicine, University of Verona, Verona, Italy; ^21^Allergy Unit and Asthma Center, Verona University Hospital, Verona, Italy; ^22^Immunoallergology Unit, Careggi University Hospital, Florence, Italy; ^23^Medineos Observational Research - An IQVIA Company, Modena, Italy; ^24^Medical Evidence R&I, AstraZeneca, Milano, Italy; ^25^Medical Affairs R&I, AstraZeneca, Milano, Italy; ^26^UO Pneumologia e Pneumologia Riabilitativa, ICS Maugeri, IRCCS Bari, Bari, Italy; ^27^Department of Medical Sciences and Public Health, University of Cagliari, Cagliari, Italy

**Keywords:** severe eosinophilic asthma, chronic rhinosinusitis with nasal polyps, benralizumab, observational, biologics

## Abstract

**Background:**

Severe eosinophilic asthma (SEA) in the presence of chronic rhinosinusitis with nasal polyps (CRSwNP) indicates the presence of a more extensive eosinophilic inflammation. *Post-hoc* analyses from a pivotal clinical trial have demonstrated the enhanced efficacy of benralizumab on asthma outcomes in patients with CRSwNP as a comorbidity.

**Methods:**

This is a *post-hoc* analysis from the Italian multi-center observational retrospective ANANKE study. Patients were divided into two groups based on self-reported CRSwNP. Baseline clinical and laboratory features in the 12 months prior to benralizumab prescription were collected. Data of change over time of blood eosinophils, annualized exacerbations rates (AER), asthma control, lung function, oral corticosteroids (OCS) use, and benralizumab discontinuation were collected during the observation period.

**Results:**

At baseline, the 110 patients with CRSwNP were less frequently female (50.9% vs 74.2%) and obese (9.1% vs. 22.6%) with higher eosinophils (605 vs. 500 cells/mm^3^) and OCS use when compared to patients without CRSwNP. Similar reductions of AER were seen (-95.8% vs. −91.5% for any exacerbation and −99.1% vs. −92.2% for severe exacerbations in patients with and without CRSwNP, respectively). During benralizumab treatment, comorbid SEA+CRSwNP was associated with a lower risk of any exacerbation (*p* = 0.0017) and severe exacerbations (*p* = 0.025). After a mean ± SD exposure of 10.3 ± 5.0 months, half of the SEA+CRSwNP patients eliminated OCS use. No discontinuation for safety reasons was recorded.

**Conclusions:**

This study helped to confirm the baseline clinical features that distinguish patients with and without CRSwNP being prescribed benralizumab. Numerically enhanced OCS reduction and lower exacerbation risk were observed in patients with SEA and comorbid CRSwNP treated with benralizumab.

## Introduction

Severe asthma (SA) is a complex heterogeneous condition that affects 5 to 10% of the patients with asthma (1). Different SA phenotypes with multiple underlying endotypes have been described during the last 20 years, with severe eosinophilic asthma (SEA) being recognized as one of the most frequent, severe, and difficult-to-treat asthma subtypes (2, 3). Indeed, SEA is associated with the presence of severe exacerbations, persistent airflow limitations, long-term use of oral (or systemic) corticosteroids (OCS), and the presence of comorbidities such as chronic rhinosinusitis with nasal polyps (CRSwNP) (2).

CRSwNP affects roughly 40% of SEA patients, and its presence is associated with the severity of asthma (4, 5). Moreover, asthma in the presence of CRSwNP is usually more difficult to treat and control, with the presence of a more extensive eosinophilic inflammation in both bronchial and nasal mucosa (6, 7). In this context, clinical evidence suggests the existence of a pathophysiological continuum between upper and lower airways eosinophilic inflammation, usually referred to as the “united airways theory” or “one airway, one disease” (6, 8). *Post-hoc* analysis from a pivotal clinical trial involving benralizumab demonstrated its augmented efficacy on asthma outcomes in patients with comorbid SEA and CRSwNP (9). Some retrospective observational studies have confirmed this evidence in real-life (10–12).

This study is a *post-hoc* analysis from the ANANKE study (13) (part of the international AstraZeneca XALOC program), an Italian multi-center observational retrospective cohort study of patients suffering from severe eosinophilic asthma who started benralizumab in the Sampling Program or as per normal clinical practice in Italy. The objective of this *post-hoc* analysis is to describe clinical characteristics and the effectiveness of benralizumab in terms of exacerbation rate, asthma control, lung function parameters, reduction of OCS maintenance dosage, and blood eosinophil count following initiation of benralizumab in patients with SEA with and without the presence of self-reported CRSwNP.

## Materials and Methods

### Study Design

The design of the ANANKE study has been fully described elsewhere (13). In brief, ANANKE (ClinicalTrials.gov Identifier: NCT04272463) is an Italian multi-center observational retrospective cohort study including patients with SEA who had started benralizumab therapy as per clinical practice or within the Italian Sampling Program, activated following benralizumab approval in January 2018 and before reimbursement for further details). Patients were consecutively enrolled between December 2019 and July 2020 in 21 Italian sites. As per protocol, data collection covered a period of >15 months, i.e., 12 months prior to the index date (initiation of the treatment with benralizumab), to retrieve a restricted set of clinical data plus at least 3 months between the index date and the enrolment visit. ANANKE was performed in accordance with the principles of the Declaration of Helsinki and with the regulations and guidelines governing medical practice and ethics in Italy. Ethical approval was provided by the ethics committees/institutional review boards at each participating site.

### Study Population

Patients have been included if they met the subsequent inclusion criteria:

1. Adult patients (age ≥18 years) at the start of benralizumab treatment within the sampling program or per clinical practice (“index date”)
2. Patients with severe eosinophilic asthma requiring a stable treatment of high doses of inhaled corticosteroids and a long-acting β2 agonist ± additional asthma controller (according to a clinician's judgment)
3. Patients who started benralizumab and received at least one injection at least 3 months before enrollment, either within the sampling program or as per routine clinical practice
4. Patients who signed the informed consent and privacy form during the enrollment visit
5. Patients with hospital medical charts available from the start of benralizumab treatment within the sampling program or per clinical practice (“index date”).

Key exclusion criteria were the following:

1. Patients who, during the observation period, received benralizumab during a clinical experimental trial
2. Patients who, during the observation period, participated in studies imposing a specific patient management strategy that does not correspond to the site's normal clinical practice.

Patients were stratified into two groups according to the positive/negative past or current self-reported history of CRSwNP at the start of benralizumab treatment. Patients without available nasal polyposis status at the index date were excluded from analyses.

### Outcomes and Variables

Each patient signed the informed consent and privacy form. Data were collected from each hospital's medical charts according to clinical practice and were entered into the electronic case report form (eCRF).

The primary endpoint was to describe the clinical features of patients with and without CRSwNP as recorded at the index date and refer to the previous 12 months of benralizumab introduction into therapy. Demographics (age, sex, body mass index [BMI], comorbidities, and smoking status), asthma features (age at diagnosis and duration), laboratory features [blood eosinophil count (BEC) and total serum immunoglobulin E (IgE)], atopic status (defined as the presence of a perennial allergen sensitization demonstrated by skin prick test), lung function parameters, asthma control [defined by Asthma Control Test (ACT)] (14)], OCS use and dosage, annualized exacerbation rates for any exacerbation (defined as a physician-diagnosed clinically relevant asthma exacerbation), and severe exacerbations [defined as worsening of asthma that leads to one of the following: a) use of systemic corticosteroids for 3 days or more or a temporary increase in a stable, background dosage of oral corticosteroids; b) an emergency department or urgent care visit (<24 h) due to asthma that required systemic corticosteroids; or c) an inpatient admission to hospital (≥24 h) due to asthma] were recorded.

Secondary endpoints were the description of outcomes during benralizumab treatment between the index date (benralizumab introduction) and end of observation (EOB); when available, data at 16, 24, and 48 weeks after the index date were described. Outcomes included in this *post-hoc* analysis were as follows: (1) change over time of BEC; (2) annualized rate of any exacerbation and severe exacerbations during benralizumab treatment; (3) change over time of asthma control; (4) change over time of forced expiratory volume in the first second (FEV_1_); (5) change over time of OCS use and dosage; (6) benralizumab discontinuation and reasons for discontinuation during the observation period. These outcomes were collected and compared in patients with and without self-reported CRSwNP.

### Statistical Analysis

Statistical analysis was described in the full manuscript (13). In brief, the analyses were descriptive and carried out using mean, standard deviation (SD), median, IQR, range, and absolute and relative frequencies. The Chi-square or Fisher exact tests, when appropriate, were used to compare patients with vs. without nasal polyposis in terms of risk of an exacerbation and severe exacerbations during benralizumab treatment. The significance threshold was set to 0.05; the performed analyses were exploratory and, therefore, correction of significance level for multiple testing was not applied. The analyses were performed using SAS software v9.4 (SAS Institute, Cary, NC, USA).

## Results

### Study Population

Between December 2019 and July 2020, a total of 205 patients were recruited and resulted to be fully eligible for all the evaluations programmed in the ANANKE study. For this *post-hoc* analysis, patients were divided into two groups—one comprised patients with CRSwNP (CRSwNP^+^, *N* = 110) and one included patients without CRSwNP (No-CRSwNP, *N* = 93)—for a total of 203 patients (Table 1). Two patients were excluded from the analyses due to the absence of nasal polyposis status at the index date.

**Table 1:** Demographic, clinical, and laboratory features of the patient population.

**Characteristics at index date**			**Total population** **(*N =* 205)**	**CRSwNP^**+**^** **(*N =* 110)**	**No-CRSwNP** **(*N =* 93)**
Female (%) (*N =* 205)	Number (percentage)		126 (61.5%)	56 (50.9%)	69 (74.2%)
Age (yrs) (*N =* 205)	Mean (SD)		55.8 (13.3)	56.5 (12.6)	55.1 (13.8)
Age at diagnosis of asthma (yrs) (*N =* 203, 110, 91)	Mean (SD)		38.9 (16.7)	38.8 (16.3)	38.7 (17.1)
Duration of asthma (yrs) (*N =* 203)	Median (IQR)		12.4 (6.3–24.6)	13.7 (6.9–24.5)	11.4 (5.7–27.3)
Blood Eosinophil count (cells/mm^3^)	Median (IQR)		580 (400–850)	605 (450–810)	500 (366–910)
Total serum IgE (IU/mL) (*N =* 123, 60, 61)	Median (IQR)		289 (85–573)	254 (116–518)	294.6 (71.4–573)
Atopy (%) (*N =* 205)	Number (percentage)		85 (41.5%)	39 (35.5%)	45 (48.4%)
BMI (*N =* 205)	Class, number (percentage)	Under/Normal	70 (34.4%)	42 (38.2%)	28 (30.2%)
		Overweight	79 (38.5%)	46 (41.8%)	33 (35.5%)
		Obese	33 (16.1%)	10 (9.1%)	21 (22.6%)
		Data unknown	23 (11.2%)	12 (10.9%)	11 (11.8%)
Smoking status (*N =* 205)	Class, number (percentage)	Non-smoker	139 (67.8%)	76 (69.1%)	63 (67.7%)
		Previous smoker	59 (24.4%)	27 (24.5%)	22 (23.7%)
		Current smoker	6 (2.9%)	2 (1.8%)	4 (4.3%)
		Data unknown	10 (4.9%)	5 (4.5%)	4 (4.3%)
Pre-bronchodilator FEV_1_ (liters) (*N =* 154, 74, 70)	Mean (SD)		2.0 (0.8)	2.1 (0.8)	1.8 (0.7)
Pre-bronchodilator FEV_1_ (% predicted) (*N =* 159, 79, 71)	Mean (SD)		70.6 (21.6)	73.2 (22.3)	68.1 (21.3)
Post-bronchodilator FEV_1_ (liters) (*N =* 92, 47, 41)	Mean (SD)		2.1 (0.9)	2.3 (0.8)	1.9 (0.8)
Post-bronchodilator FEV_1_ (% predicted) (*N =* 90, 46, 40)	Mean (SD)		75.3 (22.9)	76.6 (22.1)	74.6 (23.3)
Pre-bronchodilator FVC (liters) (*N =* 148, 71, 67)	Mean (SD)		3.0 (1.0)	3.2 (1.1)	2.7 (0.9)
Pre-bronchodilator FEV_1_/FVC (ratio) (*N =* 148, 71, 67)	Mean (SD)		0.7 (0.1)	0.7 (0.1)	0.7 (0.2)
ACT score (*N =* 161, 91, 70)	Mean (SD)		14.7 (4.7)	15 (4.7)	14.3 (4.7)
OCS use	Number (percentage)		53 (25.8%)	35 (31.8%)	18 (19.3%)
OCS dose at index date (*N =* 48, 30, 18)	Mean (SD)		14.0 (10.3)	15.7 (9.2)	10.3 (7.8)
Annualized exacerbation rate (*N =* 195, 140, 55)			4.03	3.77	4.37
Annualized severe exacerbation rate (*N =* 195, 140, 55)			1.10	1.06	1.16
**Comorbidities**	Number (percentage)				
**≥1 current asthma-related condition**			103 (50.2%)	58 (52.7%)	43 (46.2%)
GERD			43 (21%)	24 (21.8%)	18 (19.4%)
Allergic conjunctivitis			28 (13.7%)	13 (11.8%)	15 (16.1%)
Allergic rhinitis			45 (22%)	21 (19.1%)	23 (24.7%)
**≥1 current OCS-related condition**			77 (37.6%)	38 (34.5%)	38 (40.9%)
Hypertension			46 (22.4%)	22 (20%)	24 (25.8%)
Osteoporosis			23 (11.2%)	16 (14.5%)	7 (7.5%)
Cataract			12 (5.9%)	8 (7.3%)	4 (4.3%)
Anxiety/Depression			11 (5.3%)	8 (7.3%)	3 (3.2%)
Type 2 Diabetes Mellitus			10 (4.9%)	4 (3.6%)	6 (6.5%)
Obstructive sleep apnea			10 (4.9%)	2 (1.8%)	7 (7.5%)
Cardiovascular disease			7 (3.4%)	2 (1.8%)	5 (5.4%)
**Other ongoing comorbidities**			35 (17.1%)	20 (18.2%)	13 (14%)

*CRSwNP^+^, patients with present or past positive history for chronic rhinosinusitis with nasal polyps; No-CRSwNP, patients with negative history of chronic rhinosinusitis with nasal polyps; BMI, body mass index; FEV_1_, forced expiratory volume in the first second; FVC, forced vital capacity; ACT, asthma control test; OCS, oral corticosteroids; SD, standard deviation; IQR, interquartile range (25–75%)*.

Female sex was prevalent in the No-CRSwNP groups (74.2% vs. 50.9%), but the two groups were comparable in terms of age at index date, age at asthma onset, duration of the disease, and smoking status. Atopy was slightly more prevalent in the No-CRSwNP (48.4% vs. 35.5%), but similar sIgE levels were found in the two groups. A BMI ≥ 30 appeared to be more prevalent in the No-CRSwNP group (22.6% vs. 9.1%). BEC was higher in the CRSwNP group (median [range], 605 [450–810] cells/mm^3^) when compared to the No-CRSwNP patients (500 [366–910] cells/mm^3^).

Absolute volume and % of predicted FEV_1_ were lower in the No-CRSwNP groups when compared to patients with CRSwNP (1.8 liters vs. 2.1liters and 68.1% vs. 73.2%, respectively). No other lung function differences were detected between the two groups.

No differences in terms of asthma control were found between the two groups. Of note, apart from the severe asthma exacerbation rates, all exacerbation rates were numerically greater in the No-CRSwNP group when compared to the CRSwNP^+^ patients (4.37 vs. 3.77 and 1.16 vs. 1.06, respectively). These results might be explained by OCS use and dosing. Indeed, CRSwNP^+^ patients were more prone to using OCS (31.8% of OCS users at index date) and at a higher dosage (mean ± SD 15.7 ± 9.2 mg) when compared to patients without CRSwNP (19.3% of OCS users with a mean ± SD dosage of 10.3 ± 7.8 mg).

Comorbidities are summarized in Table 1. OCS-related conditions, such as osteoporosis (14.5%), cataracts (7.3%), and anxiety/depression (7.2%), were observed more frequently in CRSwNP^+^ patients when compared to patients without CRSwNP. In turn, obstructive sleep apnea (OSAS) and cardiovascular disease were more frequently detected in patients without CRSwNP.

The two groups had a mean ± SD exposure of 10.3 ± 5.0 months and a median [IQR] exposure of 9.8 [6.1–13.9] months to benralizumab therapy after the index date.

### Effect of Benralizumab on Blood Eosinophil Count

As was consistent with the known mechanism of action of benralizumab, there was a near-complete depletion of peripheral eosinophils as soon as the first time point (16 weeks, median 0, IQR 0–0), and BEC remained low thereafter (24 and 48 weeks, median 0, IQR 0–0), with no differences between the two groups.

### Effect of Benralizumab on Exacerbations Rates

**Figure 1:**
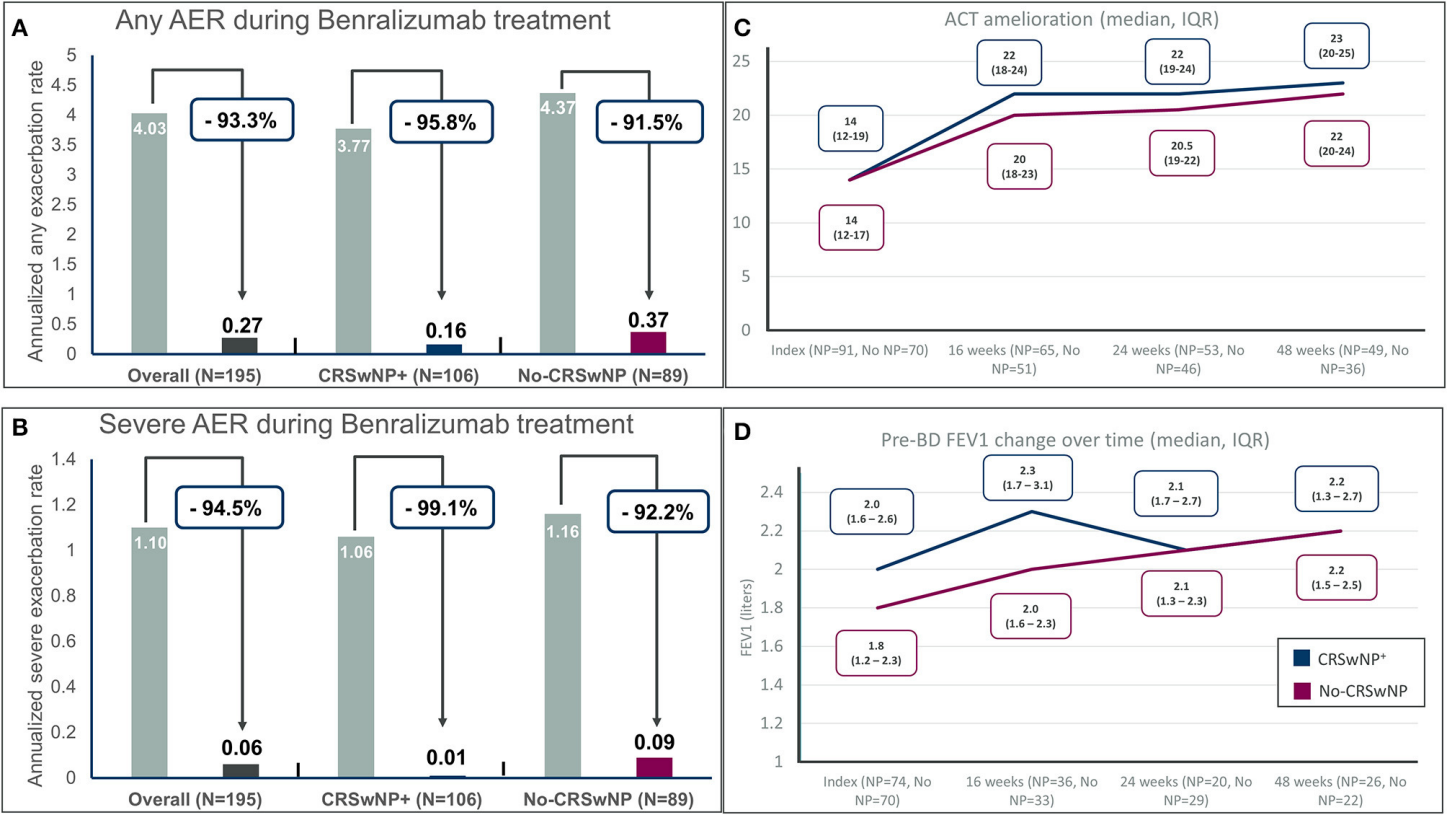
Annualized exacerbation rates (AER) of any severity **(A)** and for severe exacerbations **(B)** during benralizumab treatment in the entire population and in severe eosinophilic asthma patients with and without chronic rhinosinusitis with nasal polyps (CRSwNP); **(C)** Asthma control test (ACT) improvement in different timepoints in severe eosinophilic asthma (SEA) patients with and without chronic rhinosinusitis with nasal polyps (CRSwNP); **(D)** Pre-BD FEV1 (pre-bronchodilator forced expiratory volume in the first second) change over time in severe eosinophilic asthma (SEA) patients with and without chronic rhinosinusitis with nasal polyps (CRSwNP).

Benralizumab markedly reduced any annualized exacerbation rates in both groups, passing from 3.77 to 0.16 in CRSwNP^+^ patients (percentage of reduction, −95.8%) and from 4.37 to 0.37 in the No-CRSwNP group (reduction −91.5%) (Figure 1A). For severe exacerbations, benralizumab reduced annualized rates from 1.06 to 0.01 in patients with CRSwNP (−99.1%) and from 1.16 to 0.09 in patients without CRSwNP (−92.2%) (Figure 1B). At the end of the observation period, 89.5% of the CRSwNP patients and 71.9% in the No-CRSwNP group were exacerbation free. Notably, only one severe exacerbation was recorded in the CRSwNP^+^ group and 7 severe exacerbations were experienced in the No-CRSwNP group, none of these leading to benralizumab discontinuation at the end of follow-up. Overall, the presence of CRSwNP was associated with a lower risk of any exacerbation (*p* = 0.0017) and severe exacerbations (*p* = 0.025) in patients treated with benralizumab.

### Effect of Benralizumab on Asthma Control

Asthma control measured by ACT improved from a median of 14 (IQR 12–19 for CRSwNP^+^ and 12–17 for No-CRSwNP patients) to at least a median of 20 in both groups (Figure 1C) at 16 weeks of treatment and continued to improve till the last endpoint of 48 weeks reaching a median [IQR] of 23 [15–20] and 22 [15–19] in CRSwNP^+^ and No-CRSwNP patients, respectively. Of note, 81.6 and 80.6% of patients with and without CRSwNP, respectively, reached the cutoff of well-controlled asthma (20 points) after 48 weeks of treatment, and 76.7% of patients in both groups achieved the minimal important difference (MID) of the ACT (e.g., the improvement of ACT by at least 3 points) (15).

### Effect of Benralizumab on Lung Function

Among lung function parameters collected at baseline, sufficient data at the different time points (16, 24, and 48 weeks after benralizumab introduction) were available only for pre-bronchodilator FEV_1_ (liters). Therefore, no evaluation was made for the other lung function parameters present at baseline (predicted FEV_1_, FVC, and FEV_1_/FVC).

Figure 1D shows the sustained improvement of pre-bronchodilator FEV_1_ over time, from a median [IQR] of 2.0 liters [1.6–2.6] in the CRSwNP^+^ group and a median of 1.8 liters [1.2–2.3] in the no-CRSwNP group to a median of 2.2 liters in both groups (+200 mL and + 400 mL in the CRSwNP^+^ group and the No-CRSwNP group, respectively) after 48 weeks of treatment. The improvements were evident at the first time point recorded after 16 weeks of treatment (+300 mL for CRSwNP patients and +200 mL for patients without CRSwNP).

### Steroid-Sparing Effect of Benralizumab

**Figure 2:**
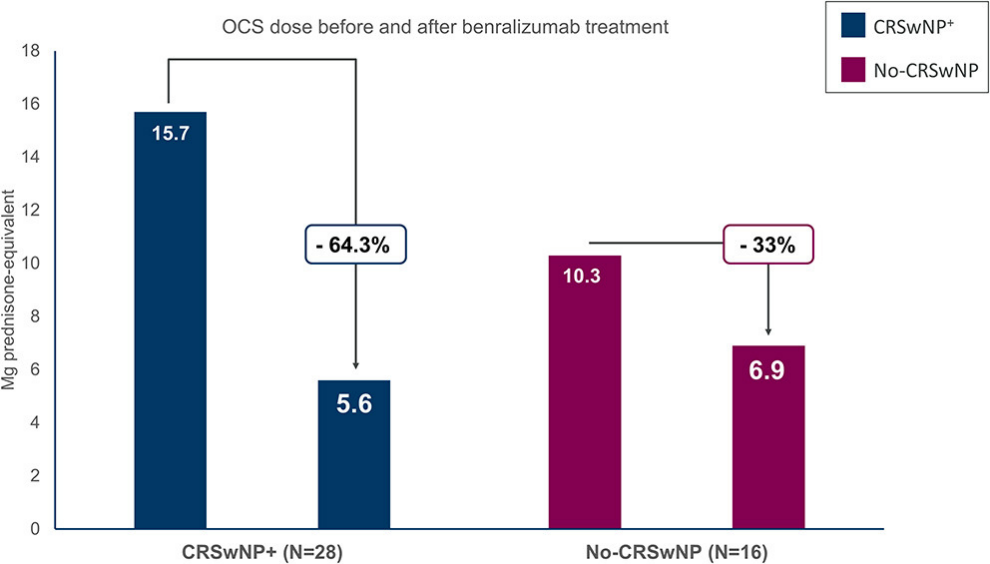
Oral corticosteroids (OCS) sparing effect of benralizumab in severe eosinophilic asthma (SEA) patients with and without chronic rhinosinusitis with nasal polyps (CRSwNP). The dose is reported in milligrams of prednisone-equivalent.

Data regarding OCS reduction and interruption during benralizumab treatment was available for 28 out of 35 and 16 out of 18 OCS users in the CRSwNP^+^ and the No-CRSwNP group, respectively (Figure 2 and Table 2).

**Table 2:** Oral corticosteroid (OCS) reduction at end of observation in the patient population.

**Variable**	**CRSwNP^**+**^ (*N =* 28)**	**No-CRSwNP (*N =* 16)**
**Reduction from baseline (%)**		
100	14 (50%)	5 (31.3%)
≥90	14 (50%)	5 (31.3%)
≥75	15 (53.6%)	5 (31.3%)
≥25	16 (57.1%)	5 (31.3%)
Any reduction	16 (57.1%)	6 (37.5%)
No reduction	12 (42.9%)	10 (53.3%)

*CRSwNP^+^, patients with present or past positive history for chronic rhinosinusitis with nasal polyps; No-CRSwNP, patients with negative history of chronic rhinosinusitis with nasal polyps; OCS, oral corticosteroids*.

In patients with CRSwNP, the mean dosage of OCS (measured in prednisone-equivalent milligrams) decreased from 15.7 ± 9.2 mg/daily to 5.6 ± 7.9 mg/daily, with a reduction of 64.3% from baseline (Figure 2). In total, 14 out of 28 patients (50%) were able to eliminate long-term OCS, and 16 out of 28 patients (57.1%) obtained a reduction in any extent of the OCS dose at EOB (Table 2).

In patients without CRSwNP, the OCS dose was reduced from a baseline of 10.3 ± 7.8 mg/daily to 6.9 ± 8.1 mg/daily at EOB (reduction −33% compared to baseline) (Figure 2). Overall, five patients (31.8%) completely discontinued OCS use and six patients accomplished a reduction of any extent of the OCS dose (37.5%) (Table 2).

### Discontinuation and Safety

Three patients discontinued benralizumab during EOB, two in the CRSwNP^+^ group and one in the No-CRSwNP group. Lack of efficacy and physician or patient decisions were the reasons recorded for discontinuation. No discontinuation for safety reasons was registered after the index date.

## Discussion

In this *post-hoc* analysis of the real-world study ANANKE (13), we evaluated the clinical features of 205 SEA patients being prescribed benralizumab in Italy according to the presence or absence of self-reported CRSwNP and we reaffirmed the efficacy of benralizumab in reducing exacerbations, OCS use, and ameliorating lung function while maintaining asthma control.

In this study, patients with self-reported CRSwNP presented clinical features in line with the known epidemiology of the disease. In greater depth, CRSwNP is reported in 40% of patients with severe asthma (4, 5) and seems to be more prevalent in men (16) with no clear association with the presence of atopy (17). A recently published paper demonstrated the link between CRSwNP, asthma, and obesity (18), but we were not able to confirm these results. However, the retrospective nature of our study may have affected this outcome.

In our study, BEC was found to be higher in patients with SEA and CRSwNP when compared to patients without (median of 605 vs. 500 cells/mm^3^, respectively). In this context, the presence of eosinophilia has been correlated with the severity of CRSwNP (19). Notably, the use of OCS has been associated with a dose-ordered suppression of BEC in patients with severe asthma (20). On the contrary, in our study, the CRSwNP^+^ group displayed higher BEC than No-CRSwNP despite more patients being treated with OCS at benralizumab introduction (31.8% vs. 19.3%) and a higher dosage (mean ± SD, 15.7 ± 9.2 mg vs. 10.3 ± 7.8 mg). These data help to confirm the eosinophilic nature of SEA in patients with CRSwNP. In this setting, the concept of United Airway Disease (UAD) helps to define the clinical-pathological link between SEA and CRSwNP and, in particular, underlines the role of eosinophils in perpetuating the chronic inflammation thus leading to airways remodeling (6, 8). Benralizumab has been able to reduce BEC to a median of zero by the first time point at 6 weeks after the first administration in both groups.

Severe asthma is often accompanied by comorbidities (21). Comorbidities do influence asthma control, and a multidisciplinary approach is useful to assess their contribution to asthma severity (22). In particular, the presence of CRSwNP in severe asthma patients has been associated with long-term OCS use (5), which, in turn, is linked with adverse events, such as cataracts and osteoporosis (23–25). In this study, the higher presence of OCS-related comorbidities in CRSwNP^+^ patients compared to patients with No-CRSwNP may be due to higher OCS exposure in the first group.

In cases of long-term OCS use or high cumulative OCS-dose exposure [e.g., > 0.5–1 gram of prednisone-equivalent per year, both as OCS bursts or long-term maintenance use (24)] in patients with severe asthma, the introduction of add-on therapy with a steroid-sparing agent can reduce exposure and therefore avoid OCS-related adverse effects (25). The ZONDA trial demonstrated the steroid-sparing efficacy of benralizumab (26), and these data have been confirmed by the 2 year integrated steroid-sparing analysis of benralizumab for severe asthma published by Bourdin and colleagues (27). In this analysis, a cumulative OCS-dose inferior to 1 gram prednisone-equivalent was reached after 1.5 years of treatment (27). In the PONENTE trial, 62.9% of SEA patients treated with benralizumab eliminated long-term OCS use, and over 80% eliminated OCS use or achieved a daily prednisone dosage of 5 mg or less if adrenal insufficiency prevented further reduction through a personalized OCS reduction algorithm (28). Despite OCs withdrawal, treatment with benralizumab was followed by improved asthma control and a drop in the exacerbation rate (28). The effect of benralizumab was independent of the presence of CRSwNP as a comorbidity of asthma (28).

The ANANKE study (13) and other real-world studies published so far (10–12, 29–31) helped to confirm these data. The present study supports previous CALIMA/SIROCCO responder analyses, where CRSwNP was identified as a clinical characteristic of enhanced response to benralizumab. Indeed, in this study half of the patients with CRSwNP have been able to eliminate OCS use after benralizumab introduction and an overall reduction of 64.3% of the OCS dose compared to the index date was observed. No statistical comparison has been made between the two groups due to the limited number of patients with retrospective data available.

In addition to the OCS-sparing effect, in this study benralizumab was able to remarkably reduce annualized asthma exacerbation rates in both patients with and without CRSwNP (−99.1% and −92.2%, respectively). In greater depth, patients with SEA and CRSwNP were at lower risk of any exacerbation and severe exacerbations when compared to the No-CRSwNP group. These data are in line with *post-hoc* analyses of SIROCCO and CALIMA pivotal clinical trials which indicated nasal polyposis as the most consistent predictor of benralizumab response in terms of asthma outcomes (9) and other real-life experiences have been able to confirm these data (11). As explained before, this enhanced efficacy could be related to the eosinophilic nature of the inflammation of both upper and lower airways present in these patients (6, 32).

Similarly, a numerically significant improvement in ACT and FEV_1_ was seen in our study in the two groups, in line with other real-life experiences (10, 11, 33). The safety profile of Benra was consistent with previous studies (34), and no new safety signals were observed.

This study does have limitations, some of them already discussed above. We acknowledge that the current or past presence of CRSwNP has been self-reported by patients at the index date or retrospectively reviewed in medical records. However, clinical and laboratory data at baseline (e.g., OCS use and BEC) help us to confirm the consistency of our data. The retrospective nature of this study can be considered a limitation but also a strength of our study, as it represents a retrospective real-world picture of the use of benralizumab in Italy with a sufficient number of patients having been included.

## Conclusions

This study helped to confirm the baseline clinical features that distinguish between patients with and without CRSwNP being prescribed benralizumab. In particular, patients with CRSwNP^+^ are less frequently female and present higher BEC at baseline despite higher OCS use frequency and dosage, though with comparable asthma control, exacerbation frequency, and lung function parameters.

Benralizumab reduced eosinophil count, exacerbation rates, and OCS use while improving asthma control and lung function in patients with and without CRSwNP. Patients with comorbid SEA and CRSwNP also experienced enhanced OCS reduction and numerically significant lower exacerbation (both any exacerbation and severe exacerbations) risk with benralizumab use.

## Data Availability Statement

The raw data supporting the conclusions of this article will be made available by the authors, on reasonable request.

## Ethics Statement

Ethical approval was provided by the Ethics Committees/Institutional Review Boards at each participating site. The patients/participants provided their written informed consent to participate in this study.

## Author Contributions

MD'A, FM, EA, EB, PB, LB, MC, GC, CC, SC, FDe, FDi, EP, GP, PR, MR, PS, GS, AV, MA, and SD: investigation, resources, and writing—review and editing. LS and AO: methodology, software, validation, formal analysis, resources, data curation, and writing—review and editing. SB: conceptualization, writing—original draft, writing—review and editing, and project administration. GV: conceptualization, writing—original draft, writing—review and editing, and visualization. All authors contributed to the article and approved the submitted version.

## Funding

Financial support for the preparation of the article was provided by AstraZeneca SpA Italy, which had a role in the study design and the collection and analysis of data.

## Conflict of Interest

FM declares research fundings as Principal investigator by AstraZeneca, Chiesi Farmaceutici, Novartis, Sanofi; fees as speaker/lecturer by AstraZeneca, Chiesi Farmaceutici, GlaxoSmithKline, Novartis, Sanofi; GC has received grant/research support from Boehringer Ingelheim, ALK, and Stallergenes, and honoraria or consultation fees from Menarini, GSK, Sanofi, Teva, Hal, AstraZeneca, and Novartis; SC declares grants and/or personal fees from AstraZeneca, Boheringer Ingelheim, Chiesi, Glaxo Smith Kline, Guidotti, Menarini, Novartis, Valeas; FDi has received lectures fees at national and international meetings and consultancy fees from Almirall, AstraZeneca, Boehringer Ingelheim, Chiesi Farmaceutici, Dompe, Guidotti/Malesci, GlaxoSmithKline, Menarini, Novartis, and Zambon; GP has received lecture fees and consultancy fees from Alfasigma, AstraZeneca, Chiesi, GlaxoSmithKline, Guidotti-Malesci, Menarini, Mundipharma, Novartis, Sanofi, Zambon; PR has participated as a lecturer, speaker, and advisor in scientific meetings and courses under the sponsorship of Almirall, AstraZeneca, Biofutura, Boehringer Ingelheim, Chiesi, GlaxoSmithKline, Menarini Group, Mundipharma, and Novartis, her department has received funding from Almirall, Boehringer Ingelheim, Chiesi, Novartis, and Zambon; MR declares grants and personal fees from Boehringer Ingelheim, Roche, AstraZeneca, Novartis, Chiesi, GSK, Menarini, Guidotti, AlfaSigma, Zambon; AV received payment for lectures and consultant arrangements from Novartis, GlaxoSmithKline, Teva, AstraZeneca; LS and AO are a employees of MediNeos Observational Research; SB and GV are AstraZeneca employees; SD received grants and/or personal fees from AstraZeneca, Chiesi, Glaxo Smith Kline, Menarini, Novartis. The remaining authors declare that the research was conducted in the absence of any commercial or financial relationships that could be construed as a potential conflict of interest.

## Publisher's Note

All claims expressed in this article are solely those of the authors and do not necessarily represent those of their affiliated organizations, or those of the publisher, the editors and the reviewers. Any product that may be evaluated in this article, or claim that may be made by its manufacturer, is not guaranteed or endorsed by the publisher.

## References

[1] Chung KF Wenzel SE Brozek JL Bush A Castro M Sterk PJ. International ERS/ATS guidelines on definition, evaluation and treatment of severe asthma. Eur Respir J. (2014) 43:343–73. 10.1183/09031936.00202013 24337046

[2] de Groot JC ten Brinke A Bel EHD. Management of the patient with eosinophilic asthma: a new era begins. ERJ Open Res. (2015) 1:24–2015. 10.1183/23120541.00024-2015 27730141 PMC5005141

[3] Heaney LG Perez de Llano L Al-Ahmad M Backer V Busby J Canonica GW. Eosinophilic and noneosinophilic asthma. Chest. (2021) 160:814–30. 10.1016/j.chest.2021.04.013 33887242

[4] Shaw DE Sousa AR Fowler SJ Fleming LJ Roberts G Corfield J. Clinical and inflammatory characteristics of the European U-BIOPRED adult severe asthma cohort. Eur Respir J. (2015) 46:1308–21. 10.1183/13993003.00779-2015 26357963

[5] Canonica GW Malvezzi L Blasi F Paggiaro P Mantero M Senna G. Chronic rhinosinusitis with nasal polyps impact in severe asthma patients: evidences from the Severe Asthma Network Italy (SANI) registry. Respir Med. (2020) 166:105947. 10.1016/j.rmed.2020.105947 32250875

[6] Ramirez GA Yacoub M-R Ripa M Mannina D Cariddi A Saporiti N. Eosinophils from physiology to disease: a comprehensive review. Biomed Res Int. (2018) 2018:1–28. 10.1155/2018/9095275 29619379 PMC5829361

[7] Laidlaw TM Mullol J Woessner KM Amin N Mannent LP. Chronic rhinosinusitis with nasal polyps and asthma. J Allergy Clin Immunol Pract. (2021) 9:1133–41. 10.1016/j.jaip.2020.09.063 33065369

[8] Passalacqua G Ciprandi G Canonica GW. United airways disease: therapeutic aspects. Thorax. (2000) 55:26S−27. 10.1136/thorax.55.suppl_2.S26 10992552 PMC1765974

[9] Bleecker ER Wechsler ME FitzGerald JM Menzies-Gow A Wu Y Hirsch I. Baseline patient factors impact on the clinical efficacy of benralizumab for severe asthma. Eur Respir J. (2018) 52:1800936. 10.1183/13993003.00936-2018 30139780 PMC6203407

[10] Menzella F Ruggiero P Galeone C Scelfo C Bagnasco D Facciolongo N. Significant improvement in lung function and asthma control after benralizumab treatment for severe refractory eosinophilic asthma. Pulm Pharmacol Ther. (2020) 64:101966. 10.1016/j.pupt.2020.101966 33039666

[11] Bagnasco D Brussino L Bonavia M Calzolari E Caminati M Caruso C. Efficacy of benralizumab in severe asthma in real life and focus on nasal polyposis. Respir Med. (2020) 171:106080. 10.1016/j.rmed.2020.106080 32917354

[12] Nolasco S Crimi C Pelaia C Benfante A Caiaffa MF Calabrese C. Benralizumab effectiveness in severe eosinophilic asthma with and without chronic rhinosinusitis with nasal polyps: a real-world multicenter study. J Allergy Clin Immunol Pract. (2021) 9:4371–80.e4. 10.1016/j.jaip.2021.08.004 34419679

[13] Menzella F Bargagli E Aliani M Bracciale P Brussino L Caiaffa MF. ChAracterization of ItaliaN severe uncontrolled Asthmatic patieNts Key features when receiving Benralizumab in a real-life setting: the observational rEtrospective ANANKE study. Respir Res. (2022) 23:36. 10.1186/s12931-022-01952-8 35183167 PMC8858449

[14] Nathan RA Sorkness CA Kosinski M Schatz M Li JT Marcus P. Development of the asthma control test?A survey for assessing asthma control. J Allergy Clin Immunol. (2004) 113:59–65. 10.1016/j.jaci.2003.09.008 14713908

[15] Schatz M Kosinski M Yarlas AS Hanlon J Watson ME Jhingran P. The minimally important difference of the Asthma Control Test. J Allergy Clin Immunol. (2009) 124:719–23.e1. 10.1016/j.jaci.2009.06.053 19767070

[16] Chen S Zhou A Emmanuel B Thomas K Guiang H. Systematic literature review of the epidemiology and clinical burden of chronic rhinosinusitis with nasal polyposis. Curr Med Res Opin. (2020) 36:1897–911. 10.1080/03007995.2020.1815682 32847417

[17] Wilson KF McMains KC Orlandi RR. The association between allergy and chronic rhinosinusitis with and without nasal polyps: an evidence-based review with recommendations. Int Forum Allergy Rhinol. (2014) 4:93–103. 10.1002/alr.21258 24395734

[18] Nam J-S Roh YH Fahad WA Noh H-E Ha J-G Yoon J-H. Association between obesity and chronic rhinosinusitis with nasal polyps: a national population-based study. BMJ Open. (2021) 11:e047230. 10.1136/bmjopen-2020-047230 34035104 PMC8154923

[19] Stevens WW Ocampo CJ Berdnikovs S Sakashita M Mahdavinia M Suh L. Cytokines in chronic rhinosinusitis. Role in eosinophilia and aspirin-exacerbated respiratory disease. Am J Respir Crit Care Med. (2015) 192:682–94. 10.1164/rccm.201412-2278OC 26067893 PMC4595675

[20] Prazma CM Bel EH Price RG Bradford ES Albers FC Yancey SW. Oral corticosteroid dose changes and impact on peripheral blood eosinophil counts in patients with severe eosinophilic asthma: a *post hoc* analysis. Respir Res. (2019) 20:83. 10.1186/s12931-019-1056-4 31053134 PMC6499981

[21] Rogliani P Sforza M Calzetta L. The impact of comorbidities on severe asthma. Curr Opin Pulm Med. (2020) 26:47–55. 10.1097/MCP.0000000000000640 31644439

[22] Boulet L-P. Influence of comorbid conditions on asthma. Eur Respir J. (2009) 33:897–906. 10.1183/09031936.00121308 19336592

[23] Sullivan PW Ghushchyan VH Globe G Schatz M. Oral corticosteroid exposure and adverse effects in asthmatic patients. J Allergy Clin Immunol. (2018) 141:110–16.e7. 10.1016/j.jaci.2017.04.009 28456623

[24] Price DB Trudo F Voorham J Xu X Kerkhof M Ling Zhi Jie J. Adverse outcomes from initiation of systemic corticosteroids for asthma: long-term observational study. J Asthma Allergy. (2018) 11:193–204. 10.2147/JAA.S176026 30214247 PMC6121746

[25] Suehs CM Menzies-Gow A Price D Bleecker ER Canonica GW Gurnell M. Expert consensus on the tapering of oral corticosteroids for the treatment of asthma. A Delphi study. Am J Respir Crit Care Med. (2021) 203:871–81. 10.1164/rccm.202007-2721OC 33112646

[26] Nair P Wenzel S Rabe KF Bourdin A Lugogo NL Kuna P. Oral glucocorticoid–sparing effect of benralizumab in severe asthma. N Engl J Med. (2017) 376:2448–58. 10.1056/NEJMoa1703501 28530840

[27] Bourdin A Shaw D Menzies-Gow A FitzGerald JM Bleecker ER Busse WW. Two-year integrated steroid-sparing analysis and safety of benralizumab for severe asthma. J Asthma. (2021) 58:514–22. 10.1080/02770903.2019.1705333 31859541

[28] Menzies-Gow A Gurnell M Heaney LG Corren J Bel EH Maspero J. Oral corticosteroid elimination via a personalised reduction algorithm in adults with severe, eosinophilic asthma treated with benralizumab (PONENTE): a multicentre, open-label, single-arm study. Lancet Respir Med. (2021) 10:47–58. 10.1016/S2213-2600(21)00352-0 34619104

[29] Pelaia C Busceti MT Crimi C Carpagnano GE Lombardo N Terracciano R. Real-Life effects of benralizumab on exacerbation number and lung hyperinflation in atopic patients with severe eosinophilic asthma. Biomed Pharmacother. (2020) 129:110444. 10.1016/j.biopha.2020.110444 32593131

[30] Pelaia C Crimi C Benfante A Caiaffa MF Calabrese C Carpagnano GE. Therapeutic effects of benralizumab assessed in patients with severe eosinophilic asthma: real-life evaluation correlated with allergic and non-allergic phenotype expression. J Asthma Allergy. (2021) 14:163–73. 10.2147/JAA.S297273 33654413 PMC7910091

[31] Kavanagh JE Hearn AP Dhariwal J D'Ancona G Douiri A Roxas C. Real-World effectiveness of benralizumab in severe eosinophilic asthma. Chest. (2021) 159:496–506. 10.1016/j.chest.2020.08.2083 32882249

[32] Ediger D Sin BA Heper A Anadolu Y Mitoasitoarlitoagil Z. Airway inflammation in nasal polyposis: immunopathological aspects of relation to asthma. Clin Exp Allergy. (2005) 35:319–26. 10.1111/j.1365-2222.2005.02194.x 15784110

[33] Scioscia G Carpagnano GE Quarato CMI Lacedonia D Santamaria S Soccio P. Effectiveness of benralizumab in improving the quality of life of severe eosinophilic asthmatic patients: our real-life experience. Front Pharmacol. (2021) 12:631660. 10.3389/fphar.2021.631660 33679414 PMC7928350

[34] Korn S Bourdin A Chupp G Cosio BG Arbetter D Shah M. Integrated safety and efficacy among patients receiving benralizumab for up to 5 years. J Allergy Clin Immunol Pract. (2021) 203:A1205. 10.1164/ajrccm-conference.2021.203.1_MeetingAbstracts.A1205 34487870

